# Fair and Effective Policing for Neighborhood Safety: Understanding and Overcoming Selection Biases

**DOI:** 10.3389/fdata.2021.787459

**Published:** 2021-11-24

**Authors:** Weijeiying Ren, Kunpeng Liu, Tianxiang Zhao, Yanjie Fu

**Affiliations:** ^1^ Computer Science Department, University of Central Florida, Orlando, FL, United States; ^2^ College of Information Sciences and Technology, The Pennsylvania State University, University Park, PA, United States

**Keywords:** counterfactual learning, neighborhood safety, fairness, stop-and-frisk, adversarial learning

## Abstract

An accurate crime prediction and risk estimation can help improve the efficiency and effectiveness of policing activities. However, reports have revealed that biases like racial prejudice could exist in policing enforcement, and trained predictors may inherit them. In this work, we study the possible reasons and countermeasures to this problem, using records from the New York frisk and search program (NYCSF) as the dataset. Concretely, we provide analysis on the possible origin of this phenomenon from the perspective of risk discrepancy, and study it with the scope of selection bias. Motivated by theories in causal inference, we propose a re-weighting approach based on propensity score to balance the data distribution, with respect to the identified treatment: search action. Naively applying existing re-weighting approaches in causal inference is not suitable as the weight is passively estimated from observational data. Inspired by adversarial learning techniques, we formulate the predictor training and re-weighting as a min-max game, so that the re-weighting scale can be automatically learned. Specifically, the proposed approach aims to train a model that: 1) able to balance the data distribution in the searched and un-searched groups; 2) remain discriminative between treatment interventions. Extensive evaluations on real-world dataset are conducted, and results validate the effectiveness of the proposed framework.

## 1 Introduction

As part of law enforcement, policing is initially expected to protect citizens, fight crime, and maintain community safety effectively. However, latent race prejudice of decision makers could adjust it towards unfair directions and impair its efficiency. As one example, the New York City police department launched *Stop-and-Frisk* (NYCSF) program, which is a policing practice of temporarily detaining, questioning, stopping civilians, and searching drivers on the street for weapons and other contraband. Such a program aims to provide communities with great potential to reduce crime in advance and alleviate social conflicts. However, an analysis Gelman et al. (2007) in NYCSF revealed that the rate of innocent people being stopped and searched is disproportionately high for those that were black or Latino. It can be seen from this case that racial bias poses great obstacles for efficient policing and resource allocation. This motivates us to 1) monitor and shape stop-and-frisk practices through data analysis and understand how the racial prejudge influence the policing system; 2) develop a debiasing solution for NYCSF program, which not only accounts for such bias, but also eases the burden on the police system and mitigates ethical conflict.

Previous works Meares (2014); Tyler et al. (2014) on NYCSF program mainly discuss the existence of biases, e.g., race, age, geographic distribution Gelman et al. (2007) and evaluate its social impact in a data analysis way. To obtain an effective countermeasure, it is promising to adopt fairness methodology Locatello et al. (2019); Zafar M. B. et al. (2017); Viswesvaran and Ones (2004); Hashimoto et al. (2018) from the machine learning community into this specific task. The main idea is to define sensitive attributes first, like race in this case, and then enforce fairness across racial groups. Recently, Khademi et al. (2019) introduces a matching-based causal fairness method and enforces predicted crime rate to be the same across race populations. However, this assumption may be too presumptuous since crime rates among race populations are different in the real-world scenario. The prompted fairness will inevitably damage the community’s safety. This observation inspires us to think: how should we understand the bias in NYCSF programs and how can we mitigate such bias without making unrealistic assumptions?

**FIGURE 1:**
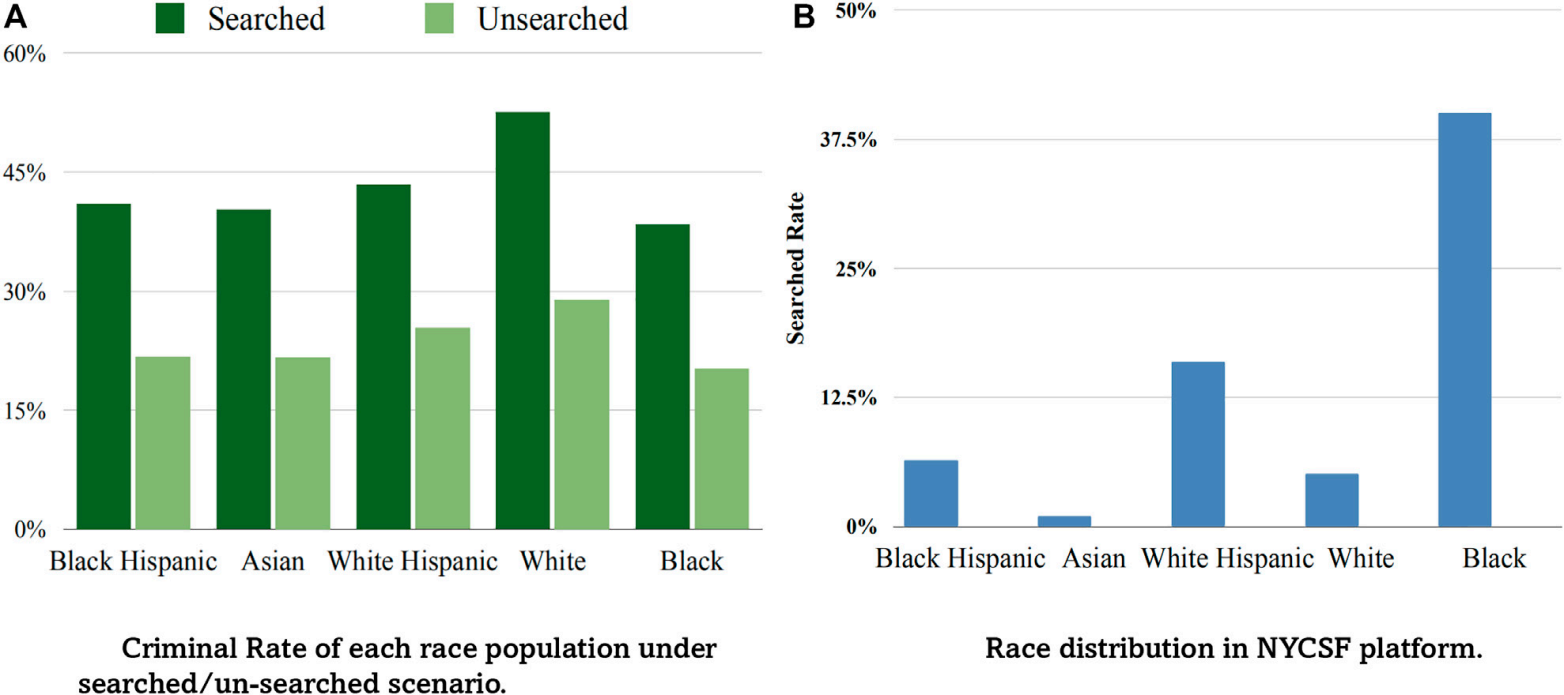
Statistical analysis on NYCSF dataset. Figure **(A)** shows the black racial population has the lowest criminal rate than other race populations in both searched/un-searched groups. Figure **(B)** shows the black is overwhelmingly searched. Selective enforcement makes more black people searched by police while most of them are innocent.

To identify the racial prejudice of polices, we conduct a series of data analysis experiments, and found that it can be modeled in the form of selection bias. Concretely, we separate each racial population into multiple groups using multiple different criteria, and compare the criminal rate in each group. From the result, we found that 1) the black race sample has the lowest criminal rate which contradicts the stereotype image of most people Gelman et al. (2007), 2) racial distribution of the observed searched group is quite different from that of the un-searched group, We visualize the results in Figure 1A. These interesting observations motivate us to study the bias embedded in the ‘search’ action. Taking one more step, we calculate the search rate of each race in the whole population, and show it in Figure 1B. It is clear that black race is overwhelmingly searched compared to other groups, hence inducing a lower criminal rate. From analyzing these statistics, one mechanism from which the bias in NYCSF program originates can be exposed: police stop and search suspicious passengers based on their own judgement, in which racial prejudices could lie, and cause the selection bias problem. Hence in this task, racial prejudice can be estimated through modeling the distribution of ‘search’ action, which in turn can be used to alleviate the bias.

Based on previous analysis, in this paper, we study a debiasing crime prediction task for the NYCSF program, and attribute the bias as a selection bias. This problem is under-explored in the NYCSF program, which poses two main challenges: 1) Lacking theoretical analysis. Why is the supervised predictor biased when training on observational data? Is there any theoretical insight that can help us understand this problem and then provide guidance to mitigate selection bias? 2) Lacking unbiased data. The biased training data lacks important signals about what the unbiased data looks like. How can we model such unbiased data distribution and design our loss function?

To answer the first question, We formulate our problem from an empirical risk minimization (ERM) perspective. The bias can be formulated as the risk discrepancy between the empirical risk and the true risk, which can be solved in a re-weighting formulation. To better understand the meaning of discrepancy, inspired by counterfactual modeling Pearl et al. (2009); Pearl (2010) and causal inference Kallus (2020); Hernán and Robins (2010); Khademi et al. (2019), we hypothesize the counterfactual distribution Wu et al. (2019a) of each driver, and then show that a supervised estimator can be unbiased only when selected probability, i.e., the searched probability of drivers by the police, is known and fixed. To sum up, selection bias accounts to risk discrepancy. This conclusion can also provide us with an insight to solve the second challenge. The insight is that a better design of selection mechanism modeling that helps us to estimate the unbiased distribution from observational data may help address this problem.

Inspired by causal inference Pearl (2010), we resort to counterfactual modeling to solve the second challenge. Regarding searched/unsearched action as a treatment/control intervention Wu et al. (2019a) respectively, the core idea in causal inference is to create a pseudo population where the distributions of the treated group and control group are similar. So the outcome is independent with treatment conditional on the confounder. Confounder is race prejudged here which introduces selective enforcement. In causal inference, inverse propensity weighting (IPW) Austin (2011) is a classical re-weighting method for its simplicity and effectiveness. Propensity score represents the probability that a driver is searched by the police. However, applying this idea into the NYCSF program is not a trivial problem. First, the propensity score in IPW is fixed and partially observed from observational data. As reported, the police enforcement way is correlative with spatial information and also depends on intractable randomness, e.g., burst events, weather circumstance. It is essential to consider unknown factors into the propensity score estimation. Second, two dilemmas make the implementation challenging. 1) We assume the driver’s criminal results should not be changed if the driver is searched or would not be searched. To achieve this goal, it is desirable to balance treatment groups with control groups w.r.t. the distributions of confounder representations. 2) Data distribution in treated and control groups should be distinguishable. Intuitively, professional experience makes policies to search for potential criminal drivers.

In response to the above problem, we propose an adversarial based re-weighting method to mitigate selection bias for NYCSF crime prediction tasks. To consider unknown factors into propensity score estimation, we do not calculate the value from observational data directly. We first formulate the counterfactual distribution estimation problem, e.g., ‘what would the criminal results be if the driver has (not) been searched?’ To learn a fair data representation, we restrict the crime label from being changed when we generate the corresponding counterfactual counterpart. Consequently, we obtain a variant of the propensity score estimator which considers uncertainty. Considering the two conflicting properties inherent in debasing NYCSF task, we formulate the two desired data of handling selection bias as a minimax game. In this game, we train a base player to improve the crime classification accuracy. At this time, the re-weighting framework balances the distribution between treated and control groups. The weighting function is regarded as an adversary, which confuses the treatment discriminator. Our contribution are listed as follows:

• We study a fair and effective policing problem from a novel selection bias perspective in the NYCSF task. We provide detailed theoretical analysis to show inconsistency issues of supervised learning on observational data. To the best of our knowledge, we are the first to analyze this problem empirically and theoretically.
• Inspired by the inverse propensity weighting method (IPW) in causal inference, We propose a simple deferred re-balancing optimization procedure to apply re-weighting more effectively. The proposed counterfactual re-weighting method connects theoretical results in causal inference with crime prediction to improve the estimation efficiency. Different from fixed propensity score estimation in IPW, the proposed re-weighting score considers unknown factors with a learning function.
• Accordingly, to both balance the data distribution in the treated and control group and make the learned distribution distinguishable, we shift the re-weighting objective into a minimax game.
• We conduct extensive experiments on the realistic NYCSF dataset to validate the effectiveness of our method. Compared to the baselines, the proposed method improves crime rate efficiency. Besides, it can mitigate racial prejudice from an exposure bias perspective, taking into account both efficiency and fairness.

## 2 Related Works

Fairness These laws typically evaluate the fairness Calmon et al. (2017); Kamiran and Calders (2009); Agarwal et al. (2018); Zafar B. et al. (2017); Louizos et al. (2015) of a decision making process using two distinct notions Zafar M. B. et al. (2017): disparate treatment Krieger and Fiske (2006); Locatello et al. (2019) and disparate impact. Feldman et al. (2015); Hashimoto et al. (2018) Disparate treatment refers to intentional discrimination, where people in a protected class are deliberately treated differently. Disparate impact refers to discrimination that is unintentional. The procedures are the same for everyone, but people in a protected class are negatively affected. While disparate impact discrimination is not always illegal. These two definitions, however, are too abstract for the purpose of computation. As a result, there is no consensus on the mathematical formulations of fairness.

In general, there has been an increasing line of work to address fairness in machine leaning models, most of them can be categorised into three groups: 1) individual fairness Biega et al. (2018); Kang et al. (2020) 2) group fairness Srivastava et al. (2019); Fleurbaey (1995) 3) causality based fairness. Kallus (2020). Individual fairness expects similar individuals to have similar outcomes. It’s not easy to find a suitable distance metric. Group fairness notions require the algorithm treat different groups equally. The most commonly used group fairness notions include demographic parity Andreev et al. (2002), equal opportunity Arneson (1989), equalized odds Hardt et al. (2016) and calibration Pleiss et al. (2017). However, they only use sensitive attributes and outcome as meaningful features. The above two notions all based on passive observed data. To provide a possible way to interpret the causes of bias, causality-based fairness notions Kusner et al. (2017) are defined based on different types of causal effects, such as total effect on interventions Funk et al. (2011), direct/indirect discrimination path-specific effects Chiappa (2019), and counterfactual fairness on counterfactual effects Garg et al. (2019); Xu et al. (2019). Identifiability Avin et al. (2005) is a critical barrier for the causality-based fairness to be applied to real applications. Wu et al. (2019b) develop a constrained optimization problem for bounding the PC fairness, which is motivated by the method proposed in Balke and Pearl (1994) for bounding confounded causal effects. It is also hard to reach a consensus in terms of what the causal graph should look like and it is even harder to decide which features to use even if we have such a graph.

Propensity Scoring in causal inference Biases caused by confounders Greenland and Robins (2009) have been extensively studied in the causal inference Pearl (2010) domain, and one most popular direction addressing it is utilizing the propensity score Austin (2011). Propensity score-based methods re-weight samples from different treatment groups, to balance the distribution. After re-weighting using propensity score, the distribution of observation will be similar across treatment groups. One classical propensity scoring methods is Inverse Propensity Weighting (IPW) Austin (2011), in which the weighting score is equal to the inverse of the probability of receiving the treatment.

Our approach also follows this line of work. However, the directly computed propensity score in existing approaches may be suboptimal, as the assumption of equality across groups is too presumptuous for this task. This observation motivates our design of introducing an adversarial module.

## 3 Stop-And-Frisk by NYPD

In this section, we will first introduce the working mechanism of the Stop-and-Frisk program, and then introduce the collected data provided by the New York City Police Department (NYPD).

### 3.1 NYCSF Program

NYPD launched the Stop-and-Frisk program for recording and analyzing police officer’s regular enforcement practice. In the Stop-and-Frisk program, there are generally three types of actors: 1) the official police, who can stop a person and check whether there are weapons or drugs carried by the suspect, with filling out a form recording the details; 2) the suspect, who is subjected to the stops; and 3) the environment,in which the stops occur, including location illustration and time records. After an individual is stopped, officers may conduct a frisk (i.e., a quick pat-down of the person’s outer clothing) if they reasonably suspect the individual is armed and dangerous; officers may additionally conduct a search if they have probable cause of criminal activity. An officer may decide to make an arrest or issue a summons, all of which is recorded on the UF-250 form. Responses are subsequently standardized, compiled and released annually to the public.

Table.1 shows the description of the NYCSF dataset. The NYCSF dataset contains a variety of heterogeneous information about all entities. Entities of each type include various information in the form of unstructured data, such as text, and structured data, such as geo-spatial, numerical, categorical, and ordinal data.

**TABLE 1:** Summary of key information recorded on the UF-250 Stop-and-Frisk form.

Feature level	Category	Feature type	Feature	Description
Character Information	Suspect Demographic Characteristic	relevant sensitive features	Race	Category: ‘BLACK HISPANIC′, ‘WHITE’,‘ASIAN ‘, ‘WHITE HISPANIC′, ‘AMERICAN INDIAN′
			Hair Color	A brief report of hair color
			Sex	Sex of suspect: male and female
			Age	Age of all suspects: from 6 to 99
			Weight/Height	Weight/Height of Suspect
			Body build	Thin, heavy or medium of the suspect
			suspect other description	first glance description about the subject
		Event Varying	Stopped Way	Frisked, Searched or Not
			Mental Activity	Suspect’s Reflection when they are stopped, like complain, calm, nerves
			Weapon found	Whether found weapon if suspect is searched/frisked
			Drug Found	Whether found drug if suspect is searched/frisked
			Criminal Label	generally, we regard arrested and summon as guilty
			initiated stop	The way to chasing the suspect who in the car: radio run, call or others
			Work Status	Whether In Uniform
	Police Profile	others	Official Rank	Official Rank, e.g., PBM, Non
		others	Official explained stop flag	Whether the police explain the reason for the stop, yes or no
		others	Official uniform flag stop	if the police are in uniform when the stop happened, yes or no
Environment Information	Primary Stop Circumstance(s)	Location Varying	Witness Report	Brief summarization Of Witness
			Witness Report	Brief Summarization Of Witness
			Inside Or Outside	Openness Of This case
		Event Varying	Stop Duration Minutes	Exact Stop Duration Time
	Location Circumstance(s)	Static	GPS Coordinates	GPS of the stopped by location
			Precinct	Precinct of location
			Location Type	Public Housing, public Transit
			Stop Location Street Name	Corresponds With Precinct

In this paper, we mainly discuss the bias introduced by subject races, hence we view race as *the sensitive attributes*. This form records various aspects of the stop, including demographic characteristics of the suspect, the time and location of the stop, the suspected crime and the rationale for the stop (e.g., whether the suspect was wearing clothing common in the commission of a crime). One notable limitation of this dataset is that no demographic or other identifying information is available about officers. The forms were filled out by hand and manually entered into an NYCSF database until 2017, when the forms became electronic. The NYCSF reports NYCSF data in two ways: a summary report released quarterly and a complete database released annually to the public.

### 3.2 Constructed Features

For the NYCSF program, we collect features from characteristic information and environment information perspectives, and highlight the key aspects of the data on Table 1.

#### 3.2.1 Characteristic Information

The subject features can be grouped into four categories: suspect demographic characteristics, suspect physical and motion profile, police profiles. We regard the subject race as *sensitive attributes* which will be discouraged in the realistic world. We also regard other demographic characteristics like suspect description, suspect hair color, suspect eye color, body build type as *sensitive relevant attributes*.

#### 3.2.2 Environment Features

The environment features can be grouped into two groups: primary stop circumstance, like furtive movement, actions of violent crime; additional stop circumstance, e.g., stop street name, time of day.

These features are very heterogeneous, including both numerical, categorical data and also text. For consistency, we represent all features as numerics or numerical vectors Ren et al. (2018). Specifically, for the categorical data with less than 8 dimensions, such office rank, office explained stop flag, we adopt the one-hot encoding, i.e., converting a categorical variable within categories into a binary vector, in which only the value in the corresponding category is set to one and the other values are set to zero. For categorical data with more than 8 dimensions, such as geo-spatial data, i.e., location precinct, City, we use the count encoding, i.e., replacing the variables by the respective count frequencies of the variables in the dataset. For text data, such as street name, suspect description, we adopt glove Pennington et al. (2014) to convert text into vector embedding and use the means of embedding to represent the semantic information. Since location information has high correspondence with subject race Gelman et al. (2007) and can leakage subject information, we assume street name, GPS coordinates, location type and precinct as *sensitive relevant attributes*.

## 4 Preliminary

In this section, we present notations used in this work, and formally define the *stop-and-frisk* crime prediction problem.

### 4.1 Problem Formulation

We first set up the notations. Consider we have a crime prediction system with a driver’s feature set *x*_*s*_ ∈ *S* and a police set *x*_*p*_ ∈ *P* in the current environment *x*_*e*_ ∈ *E*. Let *x*_*s*_ represent subject information, like driver profile and behavior information; *x*_*p*_ represents a corresponding police officer for each driver, and *x*_*e*_ represents environment information. We set $x=\left[x_{s},x_{p},x_{e}\right]\in R^{d}$ to concatenate heterogeneous data, where *d* is the number of feature dimensions. Each driver has a binary label *t* ∈ {1, 0} to denote whether the driver is searched by the police or not. In the following, to keep consistency, we set *t* = 1 as treatment and *t* = 0 as control. The associated labels *y* ∈ {0, one} are from the observational label space *Y* to represent the crime results. Formally, the collected history recording data *D*_*n*_ can be noted as a set of triples ${\left\{\left(x_{i},t_{i},y_{i}\right)\right\}}_{1⩽i⩽|D_{n}|}$ generated from an unknown distribution *p*_*u*_(*x*, *t*, *y*) over driver’s feature-treatment-crime label space *X* × *T* × *Y*. The goal of our crime prediction framework is to learn a parametric function *f*_*θ*_: *X* × *T* → *Y* from the available historical recording *D*_*n*_ to minimize the following true risk:

$$L\left(f_{\theta }\right)=E_{P_{u}\left(x,t,y\right)}\left[\left.\delta \left(f_{\theta }\left(x,t\right),y\right)\right)\right],$$ (1)

where we denote *δ*(*f*_*θ*_(*x*, *t*), *y*) as the error function of the predicted output score with *f*_*θ*_(*x*_*i*_, *t*_*i*_) with the ground-truth label *y*. *P*_*u*_(*x*, *t*, *y*) denotes the ideal unbiased data distribution. To be specific, bias refers to selection bias here, which means the searching actions are based on the police judgement.

Since the true risk is not accessible, the learning is conducted on the historical recordings *D*_*n*_ ∼ *P*_*n*_(*x*, *t*, *y*) by optimizing the following empirical risk:

$$L\left(f_{\theta }\right)=\frac{1}{\left|D_{n}\right|}\overset{\left|D_{n}\right|}{\underset{i=1}{\sum }}\delta \left(f_{\theta }\left(x_{i},t_{i}\right),y_{i}\right),$$ (2)

Based on the law of large numbers, the PAC theory Auer et al. (1995) states that empirical risk minimization can approximate the real risk if we have sufficient training data. With training instances sampled from random trials, the large amount of collected data can approximate the real data distribution for the reason of missing at random Shpitser (2016). However, as we mentioned above, because of the police selective enforcement, selection bias is demonstrated in this *stop-and-frisk* program.

## 5 Selection Biases in Stop and Frisk Program

In this section, we first show the origin of selection bias in *stop-and-frisk* program. Then, the influence of biased training data on supervised models is analyzed. Motivated by these results, we propose to address this bias issue from the selection bias perspective, and present annotations depicting the problem in the end.

Selection bias. This issue happens as police are free to select drivers and check whether they hold weapons or drugs, and these selections are latently influenced by prior prejudices, which could be unfair towards some groups. We illustrate this phenomenon in Figure 1. (a), from which it can be seen that the selective enforcement makes the observational criminal race population fail to represent the real criminal distribution across racial groups. Selective bias can be easily understood from a risk discrepancy perspective–it skews the observational distribution *p*_*n*_(*x*, *t*, *y*) from an ideal uniform selection enforcement *p*_*u*_(*x*, *t*, *y*).

Biased training on observational data. In the previous part, we have conducted a data analysis to demonstrate that race prejudice is a confounder in *stop-and-frisk* program, which lies behind the police selective enforcement. In the following part, we first provide a theoretical analysis to show the inconsistency issue of supervised models trained on a dataset with selection biases. This issue further motivate us to answer the question: can theoretical analysis provide us with several guidelines on how to alleviate the biases?

Given observational data *x* ∈ *D*_*n*_, we let *p* ^1^(*t*) = *p*(*Y* = 1, *x*, *T* = *t*) and *p* ^−1^(*t*) = *p*(*Y* = − 1, *x*, *T* = *t*), *t* ∈ {0, 1} represent the joint distribution of positive and negative criminal result under either treatment intervention. A supervised model *f*_*θ*_ is biased when training on observational data under unknown selection mechanism *p*(*t*|*x*).

$$\begin{array}{c} \begin{array}{cc} & L_{\phi }\left(f_{\theta },p^{\left(1\right)},p^{\left(-1\right)}\right)\\ & =\underset{t\in \left\{0,1\right\}}{\sum }\phi \left(f\left(x,t;\theta \right)\right)p^{\left(1\right)}\left(t\right)+\phi \left(-f\left(x,t;\theta \right)\right)p^{\left(-1\right)}\left(t\right), \end{array} \end{array}$$ (3)

We replace *f*(*x*, *t*; *θ*) with *α* for simplicity, then:

$$\begin{array}{c} \begin{array}{cc} & \inf L_{\phi }\left(f_{\theta },p^{\left(1\right)},p^{\left(-1\right)}\right)\\ & =\underset{t\in \left\{0,1\right\}}{\sum }\underset{\alpha }{\inf}\left\{\phi \left(\alpha \right)p^{\left(1\right)}\left(t\right)+\phi \left(-\alpha \right)p^{\left(-1\right)}\left(t\right)\right\}\\ & =\underset{t\in \left\{0,1\right\}}{\sum }p^{\left(1\right)}\left(t\right)\underset{\alpha }{\inf}\left\{\phi \left(\alpha \right)+\phi \left(-\alpha \right)\frac{p^{\left(-1\right)}\left(t\right)}{p^{\left(1\right)}\left(t\right)}\right\}, \end{array} \end{array}$$ (4)

Set Δ(*μ*) = − inf _*α*_(*ϕ*(*α*) + *ϕ*(− *α* ⋅ *μ*)). As Δ(*μ*) is a convex and continue function of *μ*, we can obtain:

$$\inf L_{\phi }\left(f_{\theta },p^{\left(1\right)},p^{\left(-1\right)}\right)=\underset{t\in \left\{0,1\right\}}{\sum }p^{\left(1\right)}\left(t\right)\Delta \left(\frac{p^{\left(-1\right)}\left(t\right)}{p^{\left(1\right)}\left(t\right)}\right).$$ (5)

It is clear that Eq. (5) is the f-divergence induced by Δ, where $D_{\Delta }\left(P^{\left(1\right)}\|P^{\left(-1\right)}\right)=\int f\left(\frac{dP^{\left(1\right)}}{dP^{\left(-1\right)}}\right)dP^{\left(-1\right)}$ which measures the difference between two probability distributions *P* ^(1)^ and *P* ^(−1)^. To sum up, the optimal *f*_*θ*_ can only be achieved by solving Eq. (5), where *P* ^(1)^ and *P* ^(−1)^ should be known. It can be seen as a specific form of f-divergence. It is obvious that when *P*(*t*|*X*) is unknown, the optimal *f* ^∗^ depends on *P*(*t*|*X*) will not be recovered. The traditional supervised method can only serve as a biased estimator, since they assume selection bias as 1 for all samples.

So far we have done statistical analysis on the NYPD dataset and find the criminal rate is heavily correlated with stop and frisk rates as well as spatial information. Since it is not suitable to force group fairness on crime prediction since data analysis demonstrates that each racial population indeed has different criminal rates, we propose to address this issue from the selection bias perspective–that criminal rates are subjective, the probability that each race is exposed to police. Estimators would be biased if we do not take selection bias into consideration during its design. In the next section, we will propose our solution to this problem.

Before introducing our solution, we first give the annotations used to show the selection bias. We define *y*(*t* = 1) to be the crime label if the driver would be searched by the police. Conversely, we denote *y*(*t* = 0) as the unsearched outcome in the counterfactual world. The pair (*y*(0), *y*(1)) is the potential outcomes notation in the Rubin causal model, where selection enforcement is a “treatment” and a crime result of the associated driver is an “outcome.”

Our goal is to train a supervised model *f*_*θ*_(*x*_*i*_, *t*_*i*_) which takes the instance feature *x*_*i*_ and treatment indicator *t*_*i*_ as input. We use shorthand *f*_*θ*_(*x*_*i*_, *t*_*i*_) to denote the output score and the loss with respect to *y*_*i*_ is given by *L*_*ϕ*_(*f*_*θ*_(*x*_*i*_, *t*_*i*_), *y*_*i*_). We use *p*(*t*_*i*_|*x*\*x*_*o*_) to denote the propensity score, e.g., the probability that the current driver is under the status of treatment (searched) or control (unsearched) based on driver profile and environment information.

## 6 A General Debiasing Framework

**FIGURE 2:**
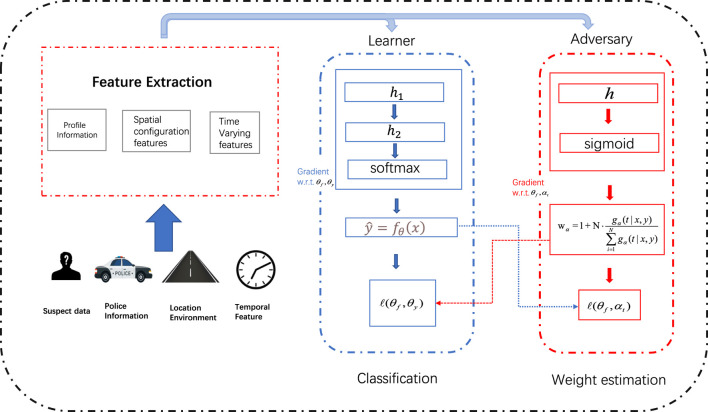
Overview of our framework. We leverage the re-weighting framework to approximate an unbiased distribution. To better estimate the re-weighting score, we estimate a counterfactual distribution in the stop and frisk program, and propose an adversarial re-weighting method. The learner aims to learn the re-weighting score and adversary aims to train the classification model.

Previous analysis shows that selection biases exist behind police enforcement, and account for the risk discrepancy. In this section, we first propose to address it via a general debiasing framework, then discuss how to formulate it inside the causal inference theory so that it can be solved with tools from that domain. In the end, we show the formalized formulation and talk about the optimization process. We illustrate our framework in Figure 2.

### 6.1 A Debiasing Empirical Risk

Data analysis has demonstrated that racial prejudice is a confounder in the NYCSF program, and causes the police selective enforcement. Motivated by it, our goal is to approximate an ideal while unknown distribution *p*_*u*_ given observational data *p*_*n*_. To deal with it, a general method is to training sample and obtain a re-weighted empirical risk function:

$$\hat{L}\left(f_{\theta }|w\right)=\frac{1}{\left|D_{n}\right|}\overset{\left|D_{n}\right|}{\underset{i=1}{\sum }}w_{i}\cdot \delta \left(f_{\theta }\left(x_{i},t_{i}\right),y_{i}\right),$$ (6)

where the weighting parameter *w*_*i*_ is properly specified, i.e., $w_{i}=\frac{p_{u}\left(x_{i},t_{i},y_{i}\right)}{p_{n}\left(x_{i},y_{i},t_{i}\right)}$ , measuring the discrepancy between selection bias eliminating data and observational data distribution. Such empirical risk is an unbiased estimation of the true risk.

As we mentioned above, selection bias arises from the fact that we only have one observational data, e.g., the polices stop and search the current data, while we known its counterfactual outcome, ‘what will the crime result be if the current driver is not searched by the police.’ To be general, we denote the counterfactual distribution as *p*_*c*_(*x*, *t*, *y*(*t*)). It is intuitive that the ideal data distribution is the combination of observational *p*_*n*_ and counterfactual data *p*_*c*_, i.e., *p*_*u*_ = *p*_*n*_ + *p*_*c*_. Since we make the assumption that whether the driver is searched or not, his crime results should not be changed. Consequently, we make the corresponding counterfactual definition:

Definition (driver-based Counterfactual outcome.) Given driver’s feature *x*, his criminal label *y*, and treatment t. We assume his potential outcome as *y*(*t*), which denotes the expected criminal label with treatment t enforced. Concretely, we assume the label should not be changed, i.e., y(t) = y.

Assumption (positivity). Given driver’s feature *x*, we assume the propensity score *p*(*t*|*x*) > 0. That is every driver has a positive probability to be searched by polices. This assumption is also consistent with the overlap assumption in causal inference.

In the following, we introduce how to model the counterfactual distribution *p*_*c*_ based on the observational data distribution *p*_*n*_.

### 6.2 Counterfactual Outcome Estimation

As we mentioned above, the re-weighted empirical risk function is an unbiased estimation of the true risk which approximates an ideal but unknown distribution, i.e., observational distribution *p*_*n*_ and counterfactual distribution *p*_*c*_. To better estimate the weighting score, we estimate the counterfactual outcome distribution in this section, and connect its formulation with re-weighting method.

In a realistic world, we only observe *y* for either *t* = 1 or *t* = 0, and the corresponding counterfactual outcome is never observed, which connects causal inference with a missing data mechanism. With re-weighting techniques, the criminal results distribution in both observational and counterfactual world can be represented as :

$$\begin{array}{cc} & p_{\alpha }\left(Y\left(t\right),T|X\right)\\ & =p\left(Y\left(t\right)|T=t,X\right)\cdot p\left(T=t|X\right)\cdot \frac{p_{\alpha }\left(T|Y\left(t\right),X\right)}{p_{\alpha }\left.\left(T=t|Y\left(t\right),X\right)\right)}, \end{array}$$ (7)

where $\frac{p_{\alpha }\left(T|Y\left(t\right),X\right)}{p_{\alpha }\left.\left(T=t|Y\left(t\right),X\right)\right)}$ counters the missing ratio in the observational distribution *p*_*n*_. To be specific, we set *T* as *t*, which makes intervention equal to observational treatment. In this case, Eq. (7) can be represented as:

$$\begin{array}{cc} & p_{\alpha }\left(Y\left(t\right),T=t|X\right)\\ & =p\left(Y\left(t\right)|T=t,X\right)\cdot p\left(T=t|X\right)\cdot \frac{p_{\alpha }\left(T=t|Y\left(t\right),X\right)}{p_{\alpha }\left.\left(T=t|Y\left(t\right),X\right)\right)}\\ & =p\left(Y\left(t\right),T=t|X\right), \end{array}$$ (8)

In which *p*_*α*_(*Y*(*t*), *T*|*X*) can be estimated from observational data. In this way, empirical risk would be equivalent to the true risk. To connect Eq. (7) with a counterfactual outcome, we set *T* = 1 − *t* and make the intervention to be the opposite action in the realistic world.

$$\begin{array}{cc} & p_{\alpha }\left(Y\left(t\right),T=1-t|X\right)\\ & =p\left(Y\left(t\right),T=t|X\right)\cdot p\left(T=t|X\right)\cdot \frac{p_{\alpha }\left(T=1-t|Y\left(t\right),X\right)}{p_{\alpha }\left.\left(T=t|Y\left(t\right),X\right)\right)}. \end{array}$$ (9)

Specifically, we set *t* = 1 in Eq. (9) and *p*_*α*_(*Y*(1), *T* = 0|*X*) represents the joint distribution that the driver is not searched by the police if he were searched. Since *p*(*Y*(*t*), *T* = *t*|*X*) and *p*(*T* = *t*|*X*) can be estimated from observational data, our goal on generate counterfactual outcome is to approximate $\frac{p_{\alpha }\left.\left(T=t|Y\left(t\right),X\right)\right)}{p_{\alpha }\left(T=1-t|Y\left(t\right),X\right)}$ including unknown factors *Y*(*t*). To ensure simplicity, we replace the term $\frac{p_{\alpha }\left.\left(T=t|Y\left(t\right),X\right)\right)}{p_{\alpha }\left(T=1-t|Y\left(t\right),X\right)}$ with a learnable function of *g*_*α*_(*T*|*X*, *Y*(*t*)). Reminding our goal in Eq. (6), we minimize the real learned risk $\hat{L}$ :

$$\begin{array}{c} J\left(\theta ,\alpha \right)≔\min\hat{L}\left(\theta ,\alpha \right)\\ =\min\frac{1}{\left|D_{n}\right|}\overset{\left|D_{n}\right|}{\underset{i=1}{\sum }}g_{\alpha }\left(T|X,Y\left(t\right)\right)\cdot \delta \left(f_{\theta }\left(x_{i},t_{i}\right),y_{i}\right). \end{array}$$ (10)

Since we do not have ground truth for *g*_*α*_(*T*|*X*, *Y*(*t*)) as supervised signals. To make Eq.(10) tractable, using definition 1, i.e., *y*(*t*) = *y*, we approximate *g*_*α*_(*T*|*X*, *Y*(*t*)) to *g*_*α*_(*T*|*X*, *Y*), which is a variant of inverse propensity score Austin (2011).

The NYCSF program can be taken as an example to illustrate the implication of *g*_*α*_(*T*|*X*, *Y*). There are two dilemmas to understand selective enforcement *p*_*α*_(*T*|*X*), where *T* ∈ {0, 1}. On the one hand, it is desirable to balance the distributions between the treated and the controlled groups, which can satisfy the unconfounded assumption in causal inference Hernán and Robins (2010):

y(1),y(0)⫫t|x. (11)

Given the latent feature representation *h*_*i*_, the representation balancing methods design a distance measurement metric, e.g., f-divergence, to minimize the representation between *P*(*h*|*x*; *t* = 1) and *P*(*h*|*x*; *t* = 0). As a consequence, the outcome is independent with treatment given the input data.

On the other hand, in the NYCSF platform, data distribution in the treated and control group should be different: criminal drivers should have a higher probability to be stopped and searched by police. In this way, the data distribution should be indicative to its treatment prediction, i.e., *P*(*h*|*x*; *t* = 1) and *P*(*h*|*x*; *t* = 0) should contain distinguish feature representation and should not be similar with each other.

With this in mind, we assume *g*_*α*_(*T*|*X*, *Y*) holding the following two properties:

• Selective Enforcement Equality. As a adjustment weight, *g*_*α*_(*T*|*X*, *Y*) is expected to balance treatment groups with control groups w.r.t, the distributions of confounder representations, e.g., race prejudge. Hence the crime results are independent with the selective enforcement.
• Selective Enforcement Disparity. At the same time, driver distributions under treatment and control are desired to be different, which is indicative of selective enforcement.

Since the aforementioned considerations contradict each other, we introduce an adversarial re-weight method for a better risk minimization.

### 6.3 Adversarial Reweighted Method

Concretely, we introduce an auxiliary *g*_*α*_ to both approximate selection-bias eliminated data distribution and make the learned distribution distinguishable to its treatment. There are many variants of generative models that meet the two requirements Creswell et al. (2018). Inspired by existing works Xu et al. (2020); Hashimoto et al. (2018), we design a min-max game to solve the above problem. Here we regard the prediction model as a player and the weighting function as an adversary for simplicity. We can formulate our fairness objective as:

$$\begin{array}{cc} J\left(\theta_{f},\theta_{y},\alpha_{t}\right)≔\underset{\theta_{f},\theta_{y}}{\min}\underset{\alpha_{t}}{\max}\hat{L}\left(\theta_{f},\theta_{y},\alpha_{t}\right)\\ & =\underset{\theta_{f},\theta_{y}}{\min}\underset{\alpha_{t}}{\max}E_{x_{i}∼D_{n}}g_{\alpha }\left(f_{\theta }\left(x\right),y;\alpha \right)\cdot \delta \left(f_{\theta }\left(x_{i},t_{i}\right),y_{i}\right)\\ & =\underset{\theta_{f},\theta_{y}}{\min}\underset{\alpha_{t}}{\max}\hat{L}\left(\theta_{f},\theta_{y}\right)/\hat{L}\left(\theta_{f},\alpha_{t}\right). \end{array}$$ (12)

To derive a concrete algorithm we need to specify how the players and adversary training parameters *θ*_*f*_, *θ*_*y*_, and *α*_*t*_. At the saddle point, the feature mapping parameters *θ*_*f*_ both minimizes crime classification loss as well as maximizes treatment prediction loss. For parameters *θ*_*y*_ and *α*_*t*_, they both penalize the crime prediction loss and treatment loss.

Observe that there is no constraint on *g*_*α*_ in Eq. (12), which makes the formulation ill-posed. Based on our Positivity assumption in causal inference, i.e, *p*(*t*|*x*) > 0, it is clear that *g*_*α*_ needs to satisfy the positivity assumption: *g*_*α*_ > 0. Besides, to prevent exploding gradients, it is important to normalize the weights across the dataset (or in the current training batch). In principle, we perform a normalization step that rescales the value of the adversary weighting component. Specifically, we make:

$$w_{\alpha }=1+N\cdot \frac{g_{\alpha }\left(t|x,y\right)}{\sum_{i=1}^{N}g_{\alpha }\left(t|x,y\right)}.$$ (13)

where *N* is current batch sizes. Finally, we present the objective function of the proposed minimax game in two stages:

$$\left({\hat{\theta }}_{f},{\hat{\theta }}_{y}\right)=\mathrm{arg min}J\left(\theta_{f},\theta_{y},{\hat{\alpha }}_{t}\right)$$ (14)

$${\hat{\alpha }}_{t}=\arg\max J\left({\hat{\theta }}_{f},{\hat{\theta }}_{y},\alpha_{t}\right).$$ (15)

Without loss of generality, we treat the objective in Eq. (12) as two component of leaner and adversary: $\arg\min_{{\hat{\theta }}_{f},{\hat{\theta }}_{y}}J\left(\theta_{f},\theta_{y},{\hat{\alpha }}_{t}\right)$ and $\arg\max_{{\hat{\alpha }}_{t}}J\left({\hat{\theta }}_{f},{\hat{\theta }}_{y},\alpha_{t}\right)$ . To handle the adversarial training, we adopt the optimization setup where the learner and adversary take turns to update their model. A more detailed training optimization can be found in Goodfellow et al. (2014).

## 7 Experiment

In this section, we first briefly describe the dataset, baselines and evaluation metrics. Then we evaluate our proposed method on the top of baselines to show its desirable performance on both the fairness and efficiency metric. Our experiments aim to answer the following research questions:

• RQ1: Can the proposed method be more robust and effective than the standard re-weighting approach, like the inverse propensity weighting method (IPW)?
• RQ2: Can the proposed method mitigate ethical conflicts in the NYCSF program as well as improve efficiency?
• RQ3: Are learned weights meaningful and why is our proposed method effective?
• RQ4: Is our method sensitive to the sensitive group size?

### 7.1 The NYC Stop and Frisk (NYCSF) Dataset

We retrieve and collect the publicly available stop, search and frisk data from The New York Police Department website during 2018 January to 2019 December. This dataset serves demographic and other information about drivers stopped by NYC police force. Since police enforcement is dynamically changed. To validate the robustness of our method, we partition the each annual data into two continues subsets, e.g., ‘NYCSF_2018F(irst)’ and ‘NYCSF_2018L(ast)’, whose durations are half years. For each subset, we select the former 4-month as training set, and the following 2 month as validation and testing set respectively. We also mix the 2 year dataset as a ‘NYCSF_Mix’ dataset, then we randomly split the dataset as 7:2:1 for training, testing and validation respectively.

For data processing, since some ‘null’ value only means ‘False’ in NYCSF stops. We carefully analyze the data, and replace ‘(null)’ with ‘F’. For the remaining data, we drop the data records with default or wrong values. For the textual feature, e.g., witness reports, we initialize the lookup table for textual data with the pre-trained vectors from GloVe Pennington et al. (2014) by setting *l* as 300. For numerical values, we encode categorical variables with one-hot embedding.

### 7.2 Baselines and Evaluation Metrics

In this section, we mainly describe baselines and evaluation metrics. For all the methods, we regard race as the sensitive attribute.

1) MLP Gardner and Dorling (1998). The vanilla model using multilayer perceptron (MLP) as the network architecture. This is the base model which does not take fairness into consideration. Since we take little attention on the model architecture in this paper and to make a fair comparison, we also use MLP as base model for other baseline.
2) IPW Austin (2011). Inverse propensity weighting (IPW) is a general re-weighting based method in causal inference which aims to balance the data distribution in treated and control groups.
3) Unawareness Grgic-Hlaca et al. (2016). Fairness through unawareness refers to leaving out sensitive attributes, such as driver race and other characteristics deemed sensitive and only takes remaining features as input.
4) PRP Hardt et al. (2016). Equality of Opportunity which is defined as an equality of the False Positive Rates across groups.
5) LFR Feng et al. (2019). LFR takes an adversarial framework to ensure that the distributions across different sensitive groups are similar.
6) Ours. We propose an adversarial re-weighting method. We train a base crime prediction classifier as a player, and use a treatment prediction classier as an adversary.

Evaluation Metric: To measure the efficiency and fairness, following existing works in Wick et al. (2019), we adopt F-1 score as our utility metric and adopt demographic parity (DP) Feng et al. (2019) as our fairness metrics. Unlike accuracy which is easy to achieve high performance for trivial predictions, F-1 shows discrepancy of class imbalance Wick et al. (2019). We stratify the test data by its race label, compute F1 score for each racial group and report: 1) Macro-F1: macro average over all racial groups; 2) Minority F-1: the lowest F1 score reported across all racial groups. To evaluate fairness, demographic parity requires the behavior of prediction models to be fair on different sensitive groups. specifically, it requires the positive rate across sensitive attributes are equal:

$$E\left(\hat{y}|S=i\right)=E\left(\hat{y}|S=j\right),\forall i\neq j$$ (16)

In the experiment, we report difference in demographic parity:

$$\Delta_{DP}=E\left(\hat{y}|S=i\right)=E\left(\hat{y}|S=j\right),\forall i\neq j$$ (17)

### 7.3 Performance Comparison

#### 7.3.1 With Inverse Propensity Weighting (IPW) Method

To answer RQ1, we fix the base classifier as MLP and conduct classification on all three datasets. To better understand if our model is effective in mitigating selection bias, we compare our method with vanilla MLP and IPW methods. Vanilla MLP does not take any strategies addressing the bias problem. IPW balances the data distribution between treated and control groups, which can minimize weighted empirical risk and approximate unbiased data distribution. We fix the base classifier as MLP and conduct classification on all five datasets.

Table 2 summarizes the main result and we make the following observations:

• The proposed model mostly achieves the best performance regarding all evaluation metrics. It manifests the importance of the adversarial re-weighting framework. Besides, selection bias in the NYCSF dataset indeed exists, hence MLP is inferior to other methods most of the time. It is interesting and essential to mitigate ethical conflicts in the NYCSF program in a debasing way.
• Selection bias, i.e., police selective enforcement, is dynamically changed with time, making it essential to take unknown factors into consideration. It is obvious that the prediction score over the all time variant dataset is different. Compared with IPW, our method is more robust since it considers unknown factors into the formulation of a dynamic weighting score. Meanwhile, it is effective to both balance treatment groups with control groups and make the data distribution distinguishable.
• IPW can improve subgroup fairness by adjusting data distribution under both treated and control groups. Compared with MLP, IPW assigns a propensity score to each sample, hence the minority is over-represented by the model–different sensitive groups have similar probability to be represented in the front of the police.

**TABLE 2:** Ours VS Inverse propensity score method.

Dataset	Method	Macro	Minority	Δ_*DP*_
		F1	F1	
NYCSF_Mix	MLP	0.9648	0.9470	0.0723
	IPW	0.9715	0.9517	0.0640
	Ours	0.9922	0.9712	0.0621
NYCSF_2018F	MLP	0.9930	0.9864	0.0848
	IPW	0.9729	0.9367	0.1072
	Ours	0.9804	0.9456	0.1039
NYCSF_2018L	MLP	0.9385	0.9129	0.0946
	IPW	0.9624	0.9229	0.1049
	Ours	0.9729	0.9401	0.0901
NYCSF_2019F	MLP	0.9221	0.8840	0.0996
	IPW	0.9763	0.9481	0.0795
	Ours	0.9922	0.9712	0.0629
NYCSF_2019L	MLP	0.9074	0.8703	0.1142
	IPW	0.9437	0.9015	0.1098
	Ours	0.9874	0.9419	0.1012

### 7.4 With Fairness Based Methods

In this section, to answer RQ2, we make a comparison with the classical fairness based methods. To make a fair comparison, we set the base classifier as a MLP for all the mentioned methods. Table 3 shows the main results, and we obtain the following observations:

• Proposed method achieves promising performance in terms of utility and fairness across all the datasets in terms of all time slot splitting. This result validates the assumption that unfairness in this task is partly due to selection biases. And our proposed method can alleviate selection for ‘stop-and-frisk’ programs.
• Fairness based constraint can improve the group fairness. While damaging their efficiency. It is obvious that fairness based baselines can eliminate group gaps, while the utility performance is lower than Ours in most of the time. This result meets the observation that directly requiring equality across racial groups is a too strong constraint and may damage its utility.
• A proper fairness notions and better model architecture design is useful to improve utility. Compared with ‘Unawareness’ and ‘PRP’ methods, LFR achieves the best utility performance, since LFR incorporates an adversarial module to enforce fairness across groups.

**TABLE 3:** Ours VS fairness based methods.

Dataset	Method	Macro	Minority	Δ_*DP*_
		F1	F1	
NYCSF_Mix	Unawareness	0.9612	0.9429	0.0718
	PRP	0.9713	0.9425	0.0629
	LFR	0.9837	0.9639	0.0598
	Ours	0.9922	0.9712	0.0621
NYCSF_2018F	Unawareness	0.9887	0.9834	0.0825
	PRP	0.9701	0.9298	0.0801
	LFR	0.9790	0.9334	0.0744
	Ours	0.9804	0.9456	0.1039
NYCSF_2018L	Unawareness	0.9311	0.9100	0.0914
	PRP	0.9598	0.9234	0.0899
	LFR	0.9712	0.9355	0.0832
	Ours	0.9729	0.9401	0.0901
NYCSF_2019F	Unawareness	0.9116	0.8813	0.0943
	PRP	0.9701	0.9455	0.0701
	LFR	0.9823	0.9630	0.0680
	Ours	0.9922	0.9712	0.0629
NYCSF_2019L	Unawareness	0.9054	0.8678	0.1125
	PRP	0.9434	0.9211	0.1022
	LFR	0.9652	0.9410	0.0983
	Ours	0.9874	0.9419	0.1012

### 7.5 Weight Analysis

**FIGURE 3:**
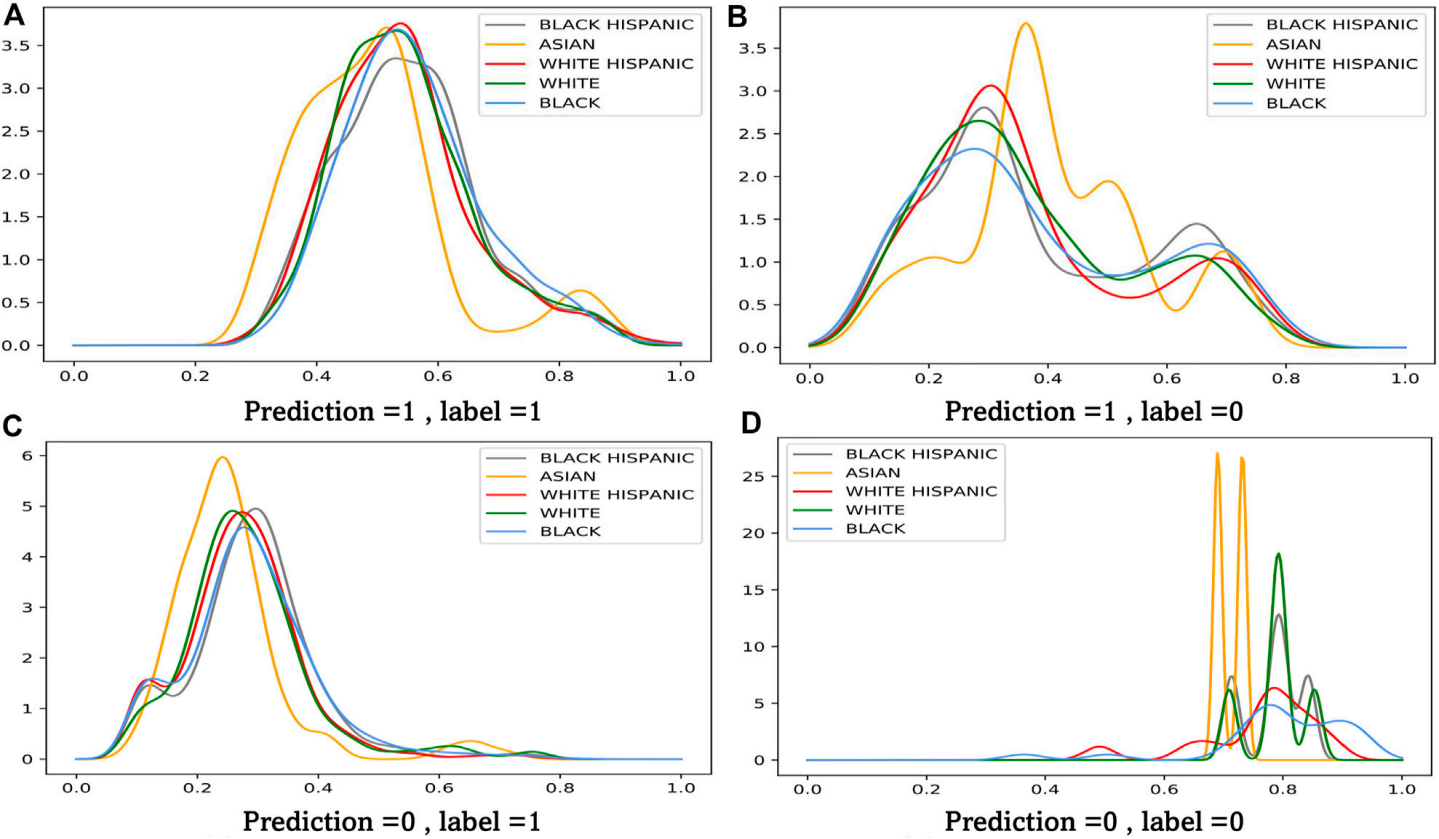
Weight Analysis of our method. **(A)** Prediction =1 , label =1 **(B)** Prediction =1 , label =0 **(C)** Prediction =0 , label =1 **(D)** Prediction =0 , label =0

We have provided a theoretical analysis on the proposed adversarial re-weighting learning method. In this section, we want to investigate RQ3, “are the learned weights really meaningful?”, directly through visualizing the example weights assigned by our model to four quadrants of a confusion matrix. The result is shown in Figure 3. Each subplot visualizes the learnt weight on *x*-axis and their corresponding density on *y*-axis. We obtain the following observations:

• Sensitive groups are upsampled with a lower inverse weighting score. It is clear that the density of minority groups, like Asian, occupies more in the low score field.
• In Figures 3A,D, samples are classified correctly. It is shown that the inverse propensity score of the ‘true positive group’ is smaller than the ‘false negative group’. Since most of the suspects are innocent, the model has more uncertainty on the ‘false positive group’. Our proposed method make a counterfactual estimation on what the suspect will do
• It is also obvious that misclassified groups are up-weighted. Comparing Figure 3B,C, most of the weight lies in the interval of [0.3,0.4]. And protected groups have more probability in this interval than the majority, like the black.

### 7.6 Sensitivity Analysis

**FIGURE 4:**
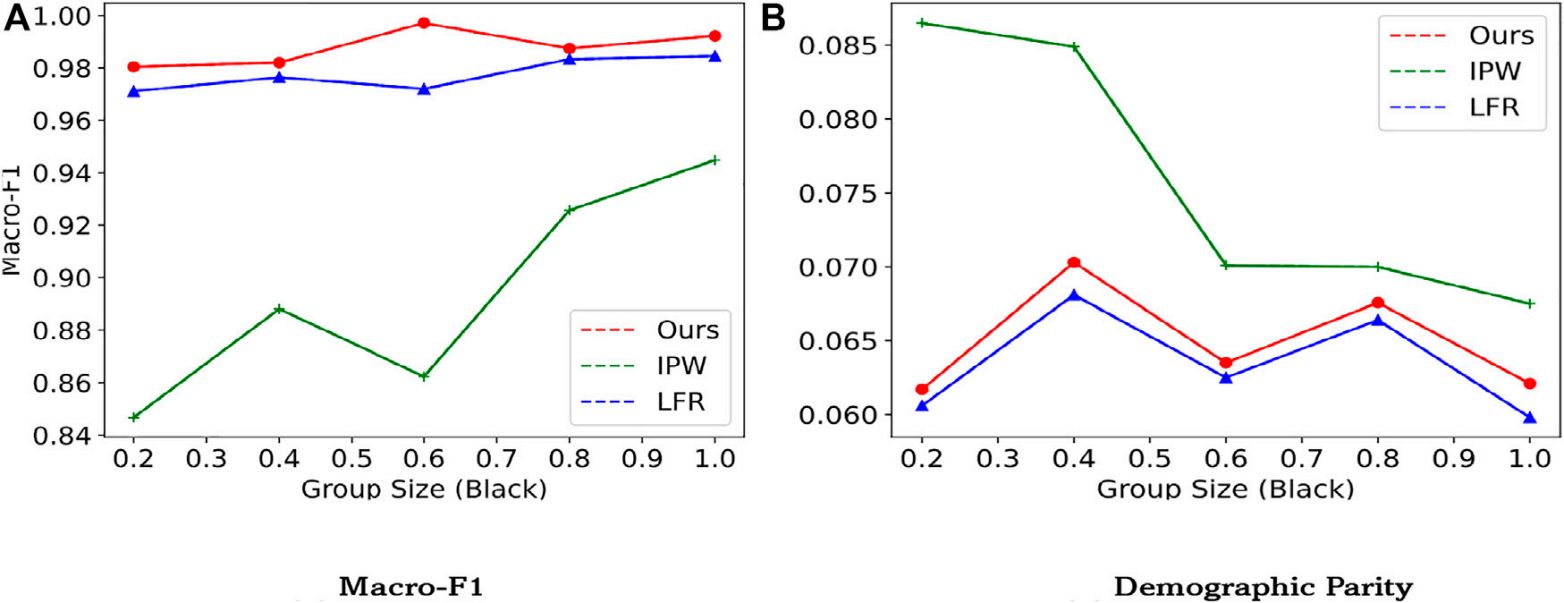
Classifier performance (F-1 score) and fairness as a function of the amount of black examples. For F-1, the higher, the better. For selection rate, the lower, the better. **(A)** Macro-F1 **(B)** Demographic Parity

In this section, to answer RQ4, we analyze the sensitivity of our proposed model towards group size, to evaluate its robustness. Selection bias indicates that different races are stopped and searched differently. To replicate selection bias, we vary the fraction of the black group and under-sample the data from 0.2 to 1.0 in the training set. We report the result on the original test dataset and show the result in Figure 4. Results are reported on the NYCSF_Mix dataset, with LFR as the baseline.

It is obvious that our model and LFR is robust to selection bias, w.r.t. the number of group sizes. As the fraction of the black race sample increases, we are forced to over-sample the black people. While the fairness metric Δ_*DP*_ for LFR keeps going up. Besides, we can also observe that IPW is sensitive towards group size. This verifies our assumption of incorporating unknown factors into the formulation of propensity score estimation.

## 8 Conclusion and Future Work

Summary. In this paper, we study the possible unfairness problem behind law enforcement using data from policing program NYCSF. Massive real-world data with detailed subject profile and environment descriptions are collected and processed as the dataset. Application Implication. Through analyzing the cause and form of biases in it, we formulate it as a selection(exposure) bias problem, and propose a countermeasure with the scope of counterfactual risk minimization. As the exposure bias is involved with unknown factors and cannot be directly measured, we design an algorithm with adversarial re-weighting, and give a detailed theoretical and experimental analysis. Future work. One future direction is to include more features in the analysis, as more behaviors and environment attribute may help learning policing practices better. Besides, modeling the dynamics of law enforcement is also one important topic, as it has been reported that the police practices evolve with time from the official website.

## Data Availability

The original contributions presented in the study are included in the article/Supplementary Material, further inquiries can be directed to the corresponding author.
